# Incomplete Data and Potential Risks of Drugs in People with Obesity

**DOI:** 10.1007/s13679-023-00532-1

**Published:** 2023-11-18

**Authors:** Caroline M. Apovian, Christopher D. Bruno, Theodore K. Kyle, Christina R. Chow, David J. Greenblatt

**Affiliations:** 1 Division of Endocrinology, Diabetes, and Hypertension, Brigham and Women’s Hospital, Harvard Medical School, Boston, MA USA; 2 Program in Pharmacology and Drug Development, Tufts University School of Medicine, Boston, MA USA; 3 Emerald Lake Safety, LLC, 23 Corporate Plaza Drive, Suite 150, Newport Beach, CA USA; 4 ConscienHealth, Pittsburgh, PA USA; 5 Clinical and Translational Science Institute, Tufts Medical Center, Boston, MA USA

**Keywords:** Obesity, Pharmacokinetics, Pharmacodynamics

## Abstract

**Purpose of Review:**

To provide examples of knowledge gaps in current pharmaceutical treatments for people with obesity and call for changes to regulatory and pharmaceutical clinical research requirements during the drug discovery and development process.

**Recent Findings:**

Treatment of obesity and its comorbidities often require the use of prescription drugs, many of which have not been fully evaluated in people with obesity. Despite a growing body of research on this topic, the impact of obesity on the pharmacokinetics and pharmacodynamics of drugs is often under-studied by drug sponsors and regulators, and subsequently underappreciated by clinicians and caretakers. There are currently multiple opportunities for pharmaceuticals to include dosing information specifically for patients with obesity in order to ensure safety and efficacy of drugs in this population. Additionally, there are serious gaps between what is known about the effects of obesity on drug disposition and the current use of drugs according to drug prescribing information and clinical practice.

**Summary:**

There is currently no requirement to test drugs in people with obesity during the drug approval process, even when preliminary data suggests there may be altered kinetics in this population. The lack of information on the safe and effective use of drugs in people with obesity may be contributing to poorer health outcomes in this population.

## Introduction

Obesity is a rapidly growing disease in the USA and around the world. First recognized as a disease by the American Medical Association in 2013, 42% of adults in the USA have obesity (BMI > 30 kg/m^2^), and 9% have severe obesity (BMI > 40 kg/m^2^) [1]. By 2030, it is projected that 49% of US adults will have obesity, and 25% will have severe obesity [2]. Globally, 13% (650 million) of adults over age 18 have obesity [3]. People with obesity have been shown to be at higher risk for health problems such as type 2 diabetes, hypertension, dyslipidemia, asthma, cancers, depression, anxiety, and schizophrenia, and obesity-related medical care costs in the USA were estimated to be $173 billion in 2019 [4]. Given the high number of comorbidities that necessitate increased medical care in people with obesity, it is critical for health care providers to be able to provide safe and effective treatment for this growing population of people.

Obesity is a multi-faceted disease with many causes, including social determinants, genetic factors, and weight gain as a side effect of some pharmaceutical treatments [5–9]. There is also a significant body of academic literature demonstrating that the pharmacokinetics of some pharmaceuticals is altered in people with obesity. In particular, the volume of distribution is increased in people with obesity for some drugs, which leads to significant increases in half-life and subsequent changes in the behavior of the drug during treatment initiation and after discontinuation [10••]. Some of the drugs that have been studied are among those that cause clinically significant weight gain, which further complicates their use in people with obesity. To address changes in drug clearance associated with body size, particularly in oncology drugs, dose strengths may be based on measures such as total body weight (TBW), ideal body weight (IBW), or body surface area (BSA) [11, 12], which are often optimized for patients of normal weight but not for patients with obesity. Despite the knowledge that obesity can change the disposition of some drugs, specific inclusion of people with obesity in clinical trials during the drug development and approval process is neither routine nor required. This disparity leads to a lack of information that would inform which treatments—and which dosing regimens—are safe and effective for use in people with obesity.

Changes in drug disposition in people with obesity and the implications of such changes for patient treatment are becoming more widely acknowledged [13–16, 17••, 18]. There have been several well-conducted studies demonstrating explicit examples in which the pharmacokinetics and pharmacodynamics of certain drugs are altered in people with obesity, yielding recommendations for changes to clinical practice. However, because the main pathway for dissemination of this information is through the academic literature, incorporation of this information into clinical practice is difficult at best. This review aims to highlight some examples of drugs where use in patients with obesity requires additional consideration, and specific recommendations for use in this population have been identified (summarized in Table 1).

**Table 1:** Examples of drugs with actionable changes for people with obesity

**Drug name**	**Common use**	**Change in people with obesity**	**Relevance for patients**	Current dosing recommendation*	**Proposed instructions for people with obesity**	**Reference**
Brexpiprazole	Anti-psychotic	Increased half-life; half-life is further increased in patients with obesity who are CYP2D6 poor metabolizers	Significantly longer time to attain effective plasma concentrations	Schizophrenia: initiate dosing at 1 mg qd for 3 days, increase to 2 mg qd for 4 days, then increase to 4 mg qd thereafter as tolerated	Schizophrenia: initiate dosing at 1 mg bd for 3 days, increase to 2 mg bd for 4 days, then increase to 4 mg qd thereafter as tolerated	[21•]
Vortioxetine	Anti-depressant	Increased half-life	Longer washout required to reduce risk of serotonin syndrome before switching to an MAOI	Wait at least 21 days after stopping vortioxetine before transitioning to an MAOI	Wait at least 31 days after stopping vortioxetine before transitioning to an MAOI	[22]
Posaconazole	Anti-fungal	Increased half-life	Risk of drug-drug interactions after stopping posaconazole	None	Require a washout period after stopping posaconazole before resuming normal CYP3A4 substrate drug administration	[26, 27]
		Lower plasma concentrations	May need increased dose for effectiveness	Monitor patients for fungal infection	For treatment of fungal infections with IV posaconazole: increase loading dose and daily maintenance dose to 400 mg in patients who weigh over 140 kg	[25•]
Micafungin	Anti-fungal	Lower plasma concentrations	May need increased dose for effectiveness	None	Increase dose to 200 mg/day in patients over 125 kg	[28, 29]
Cefazolin	Antibiotic	Lower tissue concentrations	Risk of undertreatment of infection	1–2 g IV prior to incision, with 500 mg–1 g IV every 6–8 h post-operatively	2 g IV given prior to incision, with 2 g IV every 4–6 h post-operatively	[30, 31•, 32]
Tacrolimus	Immunosuppresssant	Overdosed upon tacrolimus initiation	Risk of toxicity	Initiate dosing on a mg/kg basis (based on total body weight)	Calculate initial dose on a mg/kg basis using IBW	[33, 34•, 35]
Levonorgestrel (Plan B)	Emergency contraception	Less effective in patients over 70 kg	Higher risk of pregnancy	None	Patients over 70 kg (150 lb) should use an alternative form of emergency contraception	[36, 37, 38•]
Ibuprofen	NSAID and anti-pyretic	Higher clearance	May need increased dose for effectiveness	None	Increased doses may be necessary in patients over 70 kg	[51]

*qd* once daily, *bd* twice daily, *IBW* ideal body weight, *IV* intravenous, *MAOI* monoamine oxidase inhibitor

*As described by the package insert instructions

## Drugs with Altered Pharmacokinetics and Pharmacodynamics in Obesity

### Psychiatric Medications

**Fig. 1:**
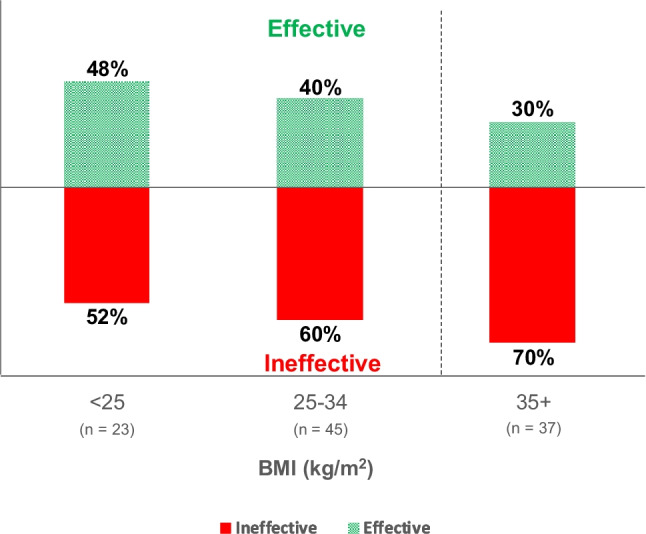
Self-reported effectiveness of brexpiprazole in patients, stratified by BMI

Brexpiprazole (Rexulti®) is an atypical antipsychotic drug that is used to treat schizophrenia, or in combination with an antidepressant to treat major depressive disorder. Patients with schizophrenia are at greater risk for obesity [19], and it is estimated that as many as 58% of patients with schizophrenia also have obesity [20]. Additionally, the product label for brexpiprazole carries a warning that its use may cause weight gain or increased cholesterol levels, both of which are concerns for people with obesity. The half-life of brexpiprazole is significantly longer in people with obesity than normal weight people, presumably due to an increase in its volume of distribution. Inasmuch as cytochrome P450-2D6 (CYP2D6) is a principal metabolic enzyme responsible for brexpiprazole clearance, patients with genetically impaired CYP2D6 metabolism may experience a further prolongation of brexpiprazole half-life. The behavior of brexpiprazole in patients with obesity was explored using physiologically-based pharmacokinetic (PBPK) modeling, which demonstrated that people with obesity may take significantly longer to reach the 90% effective concentration threshold (EC90) for brexpiprazole after initiation of treatment when compared to normal-weight patients. Additionally, modeling suggests that the subset of people with obesity who are also CYP2D6 poor metabolizers may never reach the EC90 when following the initiation protocol recommended in the package insert [21•]. A lack of efficacy during the initiation phase of brexpiprazole is concerning, as undertreatment could lead to worsening symptoms of disease, or mislead patients and clinicians to believe that the treatment failed when it was simply dosed inappropriately. In fact, preliminary results of an ongoing survey of patients who have previously taken brexpiprazole shows that patients with higher BMI are more likely to report that it was ineffective for them, with patients with BMI > 35 kg/m^2^ being most likely to report ineffective use (Fig. 1). Increasing the dose by administering brexpiprazole twice daily instead of once daily in the initiation phase—effectively giving an extended loading dose—will allow patients with obesity to attain effective concentrations in a similar timeframe as compared to those without obesity, without additional risk of side effects [21•].

Vortioxetine (Trintellix^®^) is another drug used to treat depression which has an extended half-life due to an increased volume of distribution in people with obesity [22, 23]. In this case, people with obesity appear to reach steady state concentrations in a timely manner, consistent with patients of normal weight. However, vortioxetine carries a warning that patients should not be administered a monoamine oxidase inhibitor (MAOI) within 21 days of stopping vortioxetine due to the risk of serotonin syndrome, a class-wide safety concern among serotonergic antidepressants. As the half-life of vortioxetine is approximately 50% longer in people with obesity than in normal weight people, the washout period should be extended in a corresponding manner, waiting ~ 31 days before administration of an MAOI [22] in order to ensure that people with obesity are able to transition to an MAOI with the same safety as normal weight people.

Diazepam (Valium) is a benzodiazepine commonly used to treat anxiety disorders, muscle spasms, and may also be used in patients with convulsive disorders. Its half-life during the approach to steady-state is doubled in people with obesity, and the half-life of its active metabolite desmethyldiazepam is almost four times longer compared to people without obesity (256 ± 55 h vs 69 ± 10 h, respectively) [24]. This results in an approximately doubled time to maximal drug effect during chronic dosing, which is important for patients and clinicians to recognize when initiating treatment. Likewise, the prolonged half-life also necessitates more time for diazepam and desmethyldiazepam to be fully removed from the body after discontinuation, and warrants consideration in patients who may be switching to other drugs that have similar depressive effects on the respiratory system.

### Anti-Microbials

Posaconazole is an azole anti-fungal used for the prevention or treatment of systemic fungal infections such as *Aspergillus* and *Candida*. Patients undergoing transplant or cancer treatment may be administered posaconazole as prophylaxis or to treat an infection due to their immunosuppressed state. Adequate exposure to posaconazole is necessary to effectively treat the infection, as well as to help minimize the opportunity for resistance. The posaconazole (Noxafil®) package insert briefly acknowledges that patients with weight greater than 120 kg may have lower posaconazole exposure, yet the only recommendation for these patients is to monitor closely for breakthrough infections. Wasmann et al. used modeling to show that for treatment of fungal infection, the loading dose and daily maintenance dose of intravenous posaconazole should be increased to 400 mg for individuals above 140 kg, while the standard 300 mg dose should be sufficient for prophylaxis [25•]. However, the authors are clear that these recommendations do not necessarily hold true for the oral suspension or oral delayed release tablet, and further studies are warranted for different formulations.

Furthermore, posaconazole is a known strong inhibitor of the drug metabolizing enzyme CYP3A4, and an inhibitor of the P-glycoprotein transporter. Changes in the pharmacokinetics of posaconazole in people with obesity have been shown to result in prolonged drug-drug interactions for weeks after administration of posaconazole has discontinued [26, 27]. This can be particularly dangerous, for example, in patients who are taking immunosuppressant therapy, such as the narrow therapeutic index drug tacrolimus or anti-cancer therapies that are substrates of CYP3A4. In order to minimize the risk of dangerous drug-drug interactions, a washout period of approximately 1–2 weeks should be required for patients with obesity who have stopped taking posaconazole and will be administered a CYP3A4 substrate drug. Similarly, patients with obesity who were taking a reduced dose of the CYP3A4 substrate drug concomitantly with posaconazole should also observe the same washout period before resuming the recommended monotherapy maintenance dose.

Micafungin is another anti-fungal that is important for prophylaxis and treatment of patients with *Candida* infections. Standard doses of micafungin for treatment of *Candida* infections are 100–150 mg/day. However, these doses are not sufficient to reach target concentration ratios in patients who weigh more than 125 kg [28]. Modeling shows that increasing the dose to 200 mg/day would increase the probability of reaching those target ratios in patients who weigh up to 185 kg [28, 29]. A loading dose may also help attain target ratios more quickly ^25^.

One important facet of anti-microbial therapy is maintaining a minimum inhibitory concentration (MIC), which is the lowest concentration known to be effective against the intended target. Often, MIC is measured the plasma, however, plasma concentrations do not always correlate with concentrations in the tissue where the infection resides. In patients undergoing laparoscopic surgery, concentrations of the antibiotic cefazolin are altered in the interstitial space fluid of the subcutaneous adipose tissue in patients with obesity [30], resulting in a lower probability of reaching target concentrations. This is consistent with other reports that patients with obesity are more likely to experience treatment failure and remain hospitalized for longer than patients without obesity after treatment with b-lactam antibiotics [31•], and that a higher dose of cefazolin (up to 2 g every 4–6 h) reduces the incidence of treatment failure in people with obesity [32].

### Tacrolimus

Tacrolimus is a calcineurin immunosuppressant widely used to prevent transplant rejection. With highly variable pharmacokinetics and a narrow therapeutic window, tacrolimus dosing must be optimized to prevent excessively high concentrations, which may cause nephrotoxicity, or low concentrations, leading to graft failure. As such, dosing is initiated on a mg/kg basis in an effort to attain effective concentrations in a timely manner, and therapeutic drug monitoring is an important part of patient management. Recently, studies have shown that using total body weight to calculate the initial doses of tacrolimus in renal transplant patients with obesity results in overexposure to tacrolimus [33, 34•]. A retrospective study analyzing the association between whole blood tacrolimus concentrations and various methods to calculate the therapeutic dose found that ideal body weight has a better correlation with therapeutic doses than total body weight or adjusted body weight in patients with obesity after kidney transplant [34•]. This study also found that in the first 2 weeks after transplant, patients with BMI > 35 kg/m^2^ required less tacrolimus per kilogram than patients with BMI < 25 kg/m^2^. Further, a separate retrospective study found that patients with obesity required a lower maintenance dose per kilogram than patients of normal weight at 1–6 months post-kidney transplant. At 6 months post-transplant, patients with BMI < 25 kg/m^2^ received a higher dose of tacrolimus per kilogram compared to patients with BMI > 30 kg/m^2^ after adjustments due to therapeutic drug monitoring, suggesting that dosing based on total body weight was not appropriate for these patients [35]. In total these data suggest that tacrolimus clearance does not increase in parallel with total body weight, and therefore adjusting doses based on total weight produces overexposure leading to toxicity and dose adjustments. Instead, doses based on ideal body weight appear to yield therapeutic concentrations in a timely manner and minimize the need for dose adjustments. However, package inserts for the various formulations of tacrolimus still recommend initiating dosing based on total body weight.

### Emergency Contraception

Levonorgestrel (a progestogen) is a common emergency contraception medication that is approved by the FDA for over-the-counter use. The packaging for Plan B One-Step®, the most commonly known brand of levonorgestrel emergency contraception, does not say anything about effectiveness based on bodyweight. However, Glasier et al. reported that the risk of pregnancy after taking levonorgestrel in women with obesity was more than fourfold greater compared to women with BMI < 25 kg/m^2^ (OR 4.41, 95% CI 2.05–9.44) [36]. There was also an increased risk of pregnancy after taking ulipristal acetate; however, this risk was lower (2.6-fold) than levonorgestrel [36]. It is also reported that levonorgestrel appears to be ineffective in women over 70 kg. Further research showed that women with obesity had lower concentrations (AUC and *C*_max_) of levonorgestrel compared to women with BMI < 25 kg/m^2^. Doubling the dose of levonorgestrel in women with obesity results in similar concentrations to normal weight women [37]. However, a subsequent clinical trial showed that in women with BMI > 30 kg/m^2^ and weights above 176 lb, a double dose of levonorgestrel did not improve ovulation inhibition compared to a single dose [38•]. As a result, the authors recommend that alternative emergency contraception, such as ulipristal acetate, should be used by women over 70 kg (~ 150 lb).

### Vitamin D

Vitamin D deficiency has been well-documented in people with obesity [39–44]. This deficiency is hypothesized to have a few contributing factors, including poor diet and decreased sun exposure. A lipophilic prohormone, vitamin D is subject to “volumetric dilution” in the increased adipose tissue of people with obesity [40, 42]. That is, the increased adipose tissue acts as a reservoir for vitamin D, requiring higher absolute exposure to result in the same serum vitamin D levels as people without obesity. Risks associated with vitamin D deficiency include decreased skeletal health [45] and increased cardiovascular disease [46–48]. It is unclear how vitamin D deficiency, cardiovascular disease risk, and obesity interact, but the coincidence of these disease states requires consideration. Unfortunately, it is also unclear which vitamin D supplement (vitamin D2, D3, 25-hydroxyvitamin D3, or calcidiol) and which dose strengths are most effective in increasing serum vitamin D to recommended levels in people with obesity [43, 49•]. The Endocrine Society Clinical Practice Guideline on vitamin D supplementation recommends that adults with obesity take two to three times the recommended amount of vitamin D for their age group [50]; however, the evidence to support this recommendation is scarce.

### Ibuprofen

Ibuprofen is a non-steroidal anti-inflammatory drug (NSAID) that is commonly used as an over-the-counter pain and fever reliever. It is also used for pain relief in the post-operative setting through both IV and oral administration. Research suggests that both clearance and volume of distribution are significantly different between normal-weight subjects and subjects with obesity [51]. As the observed changes to volume of distribution and clearance were parallel within individuals, the half-life of ibuprofen did not change with body composition; however, subjects with obesity had markedly lower peak plasma concentrations likely driven by more extensive distribution in this cohort. Changes in clearance were well correlated with total body weight (*R*^2^ = 0.81, *P* < 0.001), and it is worth noting that neither changes in clearance nor volume of distributions were a result of differences in plasma protein binding. Cumulatively, these data suggest that a higher dose may be necessary to achieve adequate exposure and subsequent pain control [51]. As such, the authors of this research suggest that increasing the dose strength based on excess body weight (e.g., 3.5 mg/kg above IBW) would yield more consistent exposures to patients, regardless of body size.

## Discussion and Conclusions

The examples given above are a small sampling of the work that has been done to date to better understand the effects of obesity on drug disposition and behavior. Despite this growing body of knowledge, however, uptake of this information into clinical practice has been slow. There are currently no standard processes to ensure that drugs are evaluated in people with obesity as a part of the approval process, leaving academic and clinical researchers to investigate this on their own, and with little motivation beyond scientific contribution. As a result, what is known about the effects of obesity on drug disposition is inconsistent from one drug to the next, and it is difficult to know which drugs have been adequately studied without conducting a survey of the literature. Because of this gap between the scientific literature and clinical practice, people with obesity are at risk of receiving sub-standard care.

People with obesity are generally reported to have worse outcomes for many health conditions than people without obesity. For example, BMI is an independent predictor of psychiatric hospital admission, and the authors of this study hypothesize that there may also be a relationship between coincidence of obesity and the severity of mental illnesses such as schizophrenia [52]. Additionally, people with schizophrenia and obesity are found to have increased risk of attempted suicide and more than five lifetime hospitalizations compared to people without obesity [19]. This begs the question of what treatments are effective in patients with obesity. As detailed above, changes in the pharmacokinetics of atypical antipsychotics such as brexpiprazole may lead to undertreatment of schizophrenia and further exacerbating the severity of the illness.

Heart failure patients with obesity may be placed on a left ventricular assist device (LVAD) to provide cardiac support while they lose weight in order to qualify for a transplant. However, patients with obesity and an LVAD are at higher risk of infections related to the device, and are less likely to proceed to transplant than patients without obesity [53–55]. Similarly, patients with obesity are more likely to experience surgical site infections after renal transplant compared to patients without obesity [56]. It has been proposed that patients with obesity receive prophylactic treatment to prevent infections in these situations; however, this is only a useful tool if the antimicrobials to be used are administered at an appropriate dose. Similarly, patients with obesity and bloodstream *Candida* infections require longer courses of treatment and longer hospital stays than patients without obesity [57]. People with obesity generally are at higher risk for morbidity and mortality due to nosocomial infections [58, 59]. While a physiological predisposition for infection may exist, it is also possible—if not likely—that sub-therapeutic dosing of anti-microbial agents such as those described in this paper increases the risk of treatment failure in these patients.

Many drugs, such as enoxaparin, fluconazole, and many antibiotics, are difficult to properly administer in people with obesity, and dosing changes may occur in practice which are not reflected in the product labeling for these drugs. For example, a table from a critical care textbook proposes dosing changes in people with obesity; however, these recommendations are not always reflected in the prescribing instructions and may not be standard practice (Table 2) [60]. Guidelines for how to dose anti-cancer drugs in people with obesity have recently been updated, but the quality of evidence supporting many of these recommendations is low [61]. The lack of information about how the pharmacokinetics and pharmacodynamics of drugs may or may not change in people with obesity makes treating these patients more difficult and may be contributing to poorer outcomes.

**Table 2:** Differences between practical and package insert dosing recommendations

**Drug**	**Recommendation from evidence-based critical care**		**Package insert dosing recommendation**
	**Induction**	**Maintenance**	
Lidocaine	**TBW**	**IBW**	**Dose calculated on mg/kg of TBW**
Digoxin	**IBW**	**IBW**	**Loading dose calculated using TBW; maintenance dosing calculated using LBW and renal function**
β-blockers	**IBW**	**IBW**	**Fixed dose not based on weight; titrate to effect**
Aminoglycosides	**ABW**	**ABW**	**Dose calculated on mg/kg of TBW**
Vancomycin	**ABW**	**ABW**	**Dose calculated on mg/kg of TBW and renal function**
Atracurium	TBW	TBW	Dose calculated on mg/kg of TBW
Vecuronium	**IBW**	**IBW**	**Dose calculated on mg/kg of TBW**
Phenytoin	**TBW**	**IBW**	**Fixed dose not based on weight; titrate to effect**
Corticosteroids	IBW	IBW	Variable based on disease state
Cyclosporine	**IBW**	**IBW**	**Dose calculated on mg/kg of TBW**
Aminophylline	IBW	**IBW**	Calculate loading dose based on IBW, then flat infusion rate based on age
Heparin*	**ABW**		**Fixed dose, minimum dose calculated on mg/kg of TBW**
Enoxaparin*	TBW	TBW	Dose calculated on mg/kg of TBW

Table modified from Deutschman and Neligan. Evidence-Based Practice of Critical Care (2010); package insert instructions current as of June 1, 2023. Bold indicates difference between textbook and package insert recommendations

*TBW* total body weight, *IBW* ideal body weight

ABW = IBW + 0.4*(TBW-IBW)

*For treatment of venous thromboembolism

**Fig. 2:**
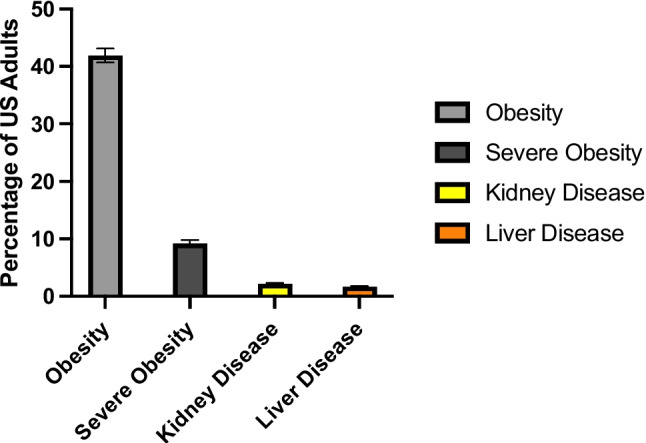
Percentage of US adults with selected chronic diseases according to the CDC [62]

It is critical to understand whether the treatments for the various comorbidities of obesity are safe and effective for these patients, as is standard for patients with renal or hepatic impairment. However, despite FDA guidance to include patients “at the extremes of weight” in clinical trials [63], there is currently no regulatory requirement or incentive for pharmaceutical developers to include patients with obesity in clinical trials during the approval of a new drug. Given that 42% percent of US adults have obesity, compared to 2.2% of adults with kidney disease and 1.7% with liver disease (Fig. 2) [62], it should be required that drug sponsors assess new drugs for the potential pharmacokinetic changes in people with obesity and, if warranted, conduct specific studies to examine the clinical relevance of such changes. In November 2022, the FDA held a workshop to discuss designating people with obesity as a special population for study during the drug approval process, signaling regulatory awareness of this issue during drug discovery and development and the need to address it. Identifying people with obesity as a special population would speed awareness of the potential for changes in drug pharmacokinetics and pharmacodynamics and may increase the likelihood that this information is taught and implemented as part of standard medical training. As it stands now, however, there is a significant body of literature demonstrating clinically relevant changes of some drugs in people with obesity. The incomplete data and potential risks of drugs in people with obesity requires attention—from clinicians, researchers, and regulators, especially in light of the potential that these gaps could be adding to the poor clinical outcomes experienced by the substantial population of people living with obesity.

## Data Availability

Not applicable.
